# Distinctive Skeletal Abnormalities With No Microdeletions or Microduplications on Array-CGH in a Boy With Mohr Syndrome (Oro-Facial-Digital Type II)

**DOI:** 10.14740/jocmr2341w

**Published:** 2015-10-23

**Authors:** Ali Al Kaissi, Renata Pospischill, Franz Grill, Rudolf Ganger

**Affiliations:** a Ludwig Boltzmann Institute of Osteology at the Hanusch Hospital of WGKK and AUVA Trauma Centre Meidling, First Medical Department, Hanusch Hospital, Vienna, Austria; b Orthopaedic Hospital of Speising, Paediatric Department, Vienna, Austria

**Keywords:** OFD type II, Mohr syndrome, Distinctive bony changes, Radiology

## Abstract

We describe a constellation of distinctive skeletal abnormalities in an 8-year-old boy who presented with the full clinical criteria of oro-facial-digital (OFD) type II (Mohr syndrome): bony changes of obtuse mandibular angle, bimanual hexadactyly and unilateral synostosis of the metacarpo-phalanges of 3-4, bilateral coxa valga associated with moderate hip subluxation, over-tubulation of the long bones, vertical talus of the left foot and talipes equinovarus of the right foot respectively. Interestingly, we encountered variable minor malformations in his parents, confirming the autosomal recessive pattern of inheritance. There were no microdeletions or microduplications after performing array-CGH-analysis. We report what might be a constellation of unreported skeletal abnormalities in a child with OFD type II (Mohr syndrome).

## Introduction

Oro-facial-digital (OFD) syndrome type I is characterized by midline cleft or notch of the upper lip, multiple hyperplastic oral frenula, asymmetrical cleft palate, clefting of the maxillary alveolar ridge into an anterior portion containing the canine teeth and two posterior segments, and a lobulated tongue with hamartomata. The facial features are prominent milia in infancy, a broad nasal root, dystopia canthorum, hypoplasia of the alar cartilages and narrow nares. The digital features are brachydactyly and skin syndactyly of the hands, clinodactyly, polydactyly of the hallux, and brachydactyly of the toes. The gene is lethal in males, who are thought to die *in utero*, although occasional, severely affected; near term cases are reported [1-4].

Mohr [5] described a family with expanded several sibs and a cousin exhibiting lobed tongue, manual polydactyly, and bilateral polysyndactyly of the halluces. There was a midline cleft of the upper lip, hypertelorism, and micrognathia, and the ears were low-set and posteriorly angulated. The bony changes were described to be limited to hands and feet (bilateral manual hexadactyly and bilateral polysyndactyly of the halluces were characteristics). Though, bimanual hexadactyly apparently is not a requisite for the diagnosis of Mohr syndrome, since in most cases, there have been five fingers with ulnar deviation of the fifth finger, 3-4 syndactyly with extra bones in the web, or hexadactyly of only one hand. Mental retardation has been reported in several cases associated with muscular hypotonia and poor coordination [6-8].

Generally speaking, OFD type II is similar to OFD type I but can usually be differentiated from it. Both males and females can be affected. The hair is normal. The tip of the nose may be bifid and milia are not pronounced in infancy. Otherwise the facial and oral features are similar to OFD I. Another distinguishing feature is conductive hearing loss in OFD type II. In the limbs, there may be post-axial polydactyly and skin syndactyly of the fingers, and bilateral duplication of the hallux is characteristic. Pseudarthrosis of the tibia can be a feature [6, 9-11].

## Case Report

### Clinical report

An 8-year-old boy was referred to our department for clinical assessment. He was a product of full term uneventful gestation. His movement *in utero* has been described by the mother as feeble and weak. At birth his length was 56 cm, weighing 4,650 g and his head circumference measured 38 cm. There were several associated anomalies noted by the pediatrician, namely a median cleft lip/cleft palate, bilateral hexadactyly of the hands and polysyndactyly of the foot associated with unilateral club foot. The mother was 27-year-old gravida 1 abortus 1 married to a 32-year-old unrelated man, but both were from the same geographical origin for generations. Family history was contributory. Both parents manifested minor oral and skeletal malformations and relatives from either side showed skeletal malformations such as club foot and minor short bones deformities relevant to the index case. The child underwent a series of corrective operations for the cleft palate, and at age of 9 months, a corrective operation was performed on his club foot. The hexadactyly of the hands have been removed thereafter.

His subsequent course of development has been of remarkable retardation in acquiring the skills of gross motor (he started to walk at age of 3 years, albeit with difficulty), speech and language retardation in connection with hearing loss and defective coordination skills were apparent. Prior to examination in our hospital, he was diagnosed as being manifesting Greig syndrome. He underwent a series of vigorous investigations. Structural chromosomal aberrations were excluded via 20 CAG-banded mitoses. Testing of the parents showed normal chromosomes. There were no microdeletions or microduplication after performing array-CGH-analysis (this was performed with 44 K-oligonucleotide-array in the GL13 gene; extended analysis of the gene showed no significant results).

**Figure 1:**
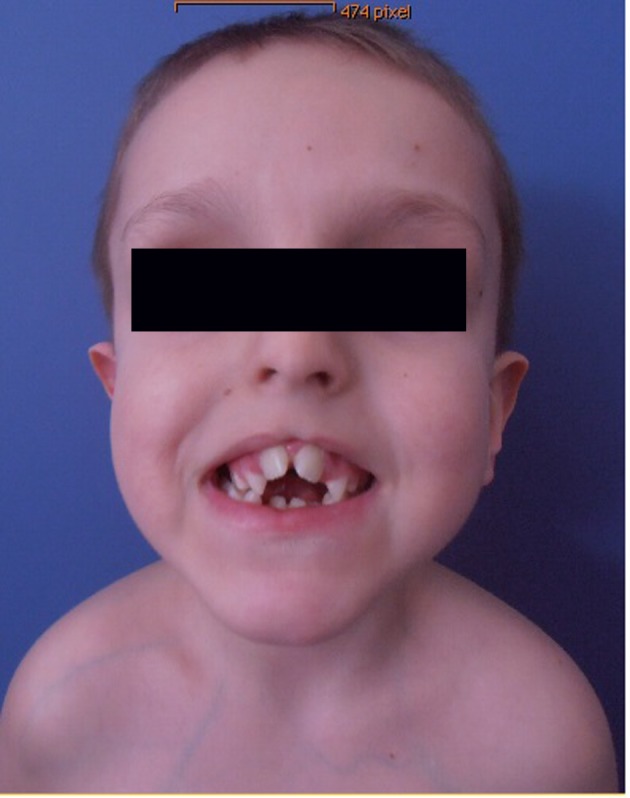
Photo showed deceleration of growth (-3 SD) and peculiar craniofacial and oral abnormalities such as sparse skull hair, frontal bossing, depressed nasal bridge and hypertelorism and epicanthic folds. Patient manifested bifid nose with hypoplasia of the alae and a short philtrum and a median partial cleft lip and an operated unilateral paramedian cleft and lobated tongue with a papilliform protuberance and hamartoma of the ventral surface of the tongue.

**Figure 2:**
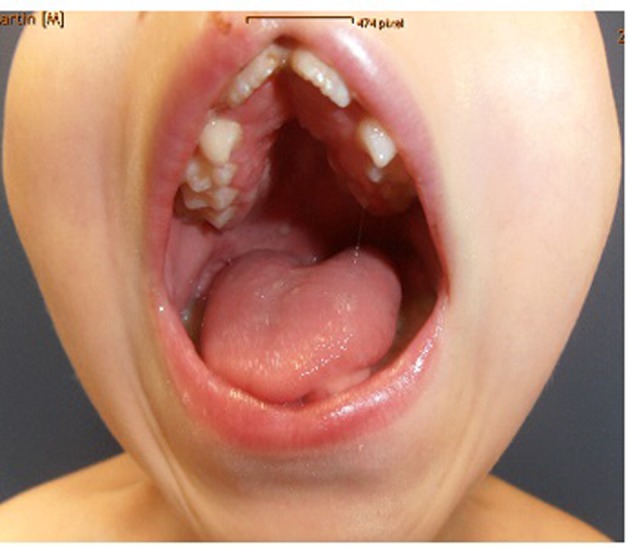
Flattening of the alveolar crest with a median sulcus and several nodules and a cleft palate associated with gingival fibromatosis, fusion of the lateral incisors (bilaterally). Ears were large, low-set and showed ill-defined modeling.

Clinical examination showed deceleration of growth (-3 SD) and peculiar craniofacial and oral abnormalities such as sparse skull hair, frontal bossing, depressed nasal bridge and hypertelorism and epicanthic folds. He manifested bifid nose with hypoplasia of the alae and a short philtrum and a median partial cleft lip and an operated unilateral paramedian cleft and lobated tongue with a papilliform protuberance and hamartoma of the ventral surface of the tongue (Fig. 1). There were flattening of the alveolar crest with a median sulcus and several nodules and a cleft palate associated with gingival fibromatosis, and fusion of the lateral incisors (bilaterally), and ears were large, low-set and showed ill-defined modeling (Fig. 2). Musculoskeletal examination showed transparent skin with apparent veins associated with moderate ligamentous hyperlaxity and muscular hypotonia, funnel chest with dysplastic nipples. The hands were short with clinodactyly of the fifth fingers and his bilateral postaxial hexadactyly were operated early on. The feet were short with duplication of the hallux (polysyndactyly). Vision and neurological examination were normal. He manifested dysarthria and conductive hearing loss.

### Skeletal survey

**Figure 3:**
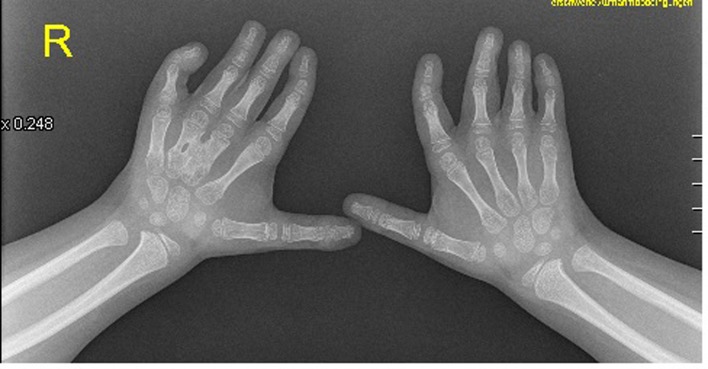
The hands were short with clinodactyly of the fifth fingers and his bilateral postaxial hexadactyly were operated early on. The feet were short with duplication of the hallux (polysyndactyly). Anteroposterior radiograph of the hands showed bilateral ulnar deviation, unilateral synostosis of the right third and fourth metacarpals, and marked dysplasia of the intermediate phalanges bilaterally.

**Figure 4:**
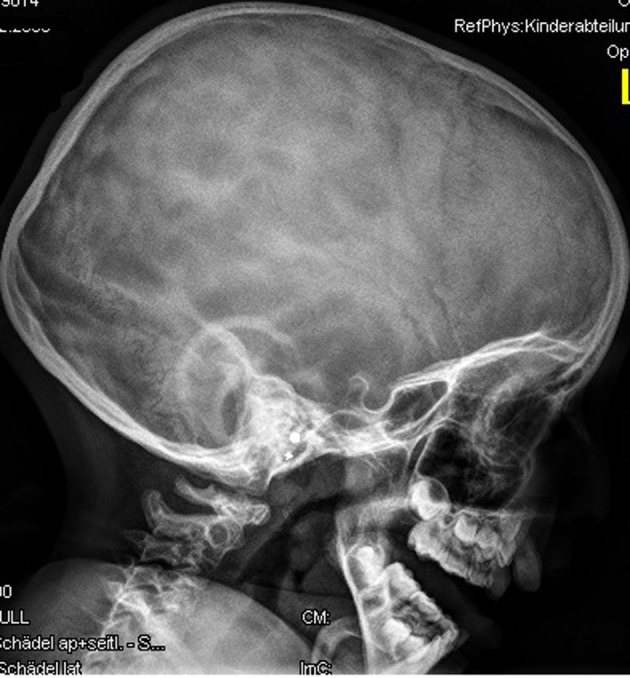
Lateral skull radiograph showed obtuse angle of the mandible, wormian bones, hypoplasia of the zygoma, mandible and maxilla respectively.

Anteroposterior radiograph of the hands showed bilateral ulnar deviation, unilateral synostosis of the right third and fourth metacarpals, and marked dysplasia of the intermediate phalanges bilaterally (Fig. 3). Lateral skull radiograph showed obtuse angle of the mandible, wormian bones, hypoplasia of the zygoma, mandible and maxilla respectively (Fig. 4).

Lateral spine radiograph showed mild scalloping of the posterior endplates along the lumbar vertebrae and loss of the physiological lumbar lordosis.

**Figure 5:**
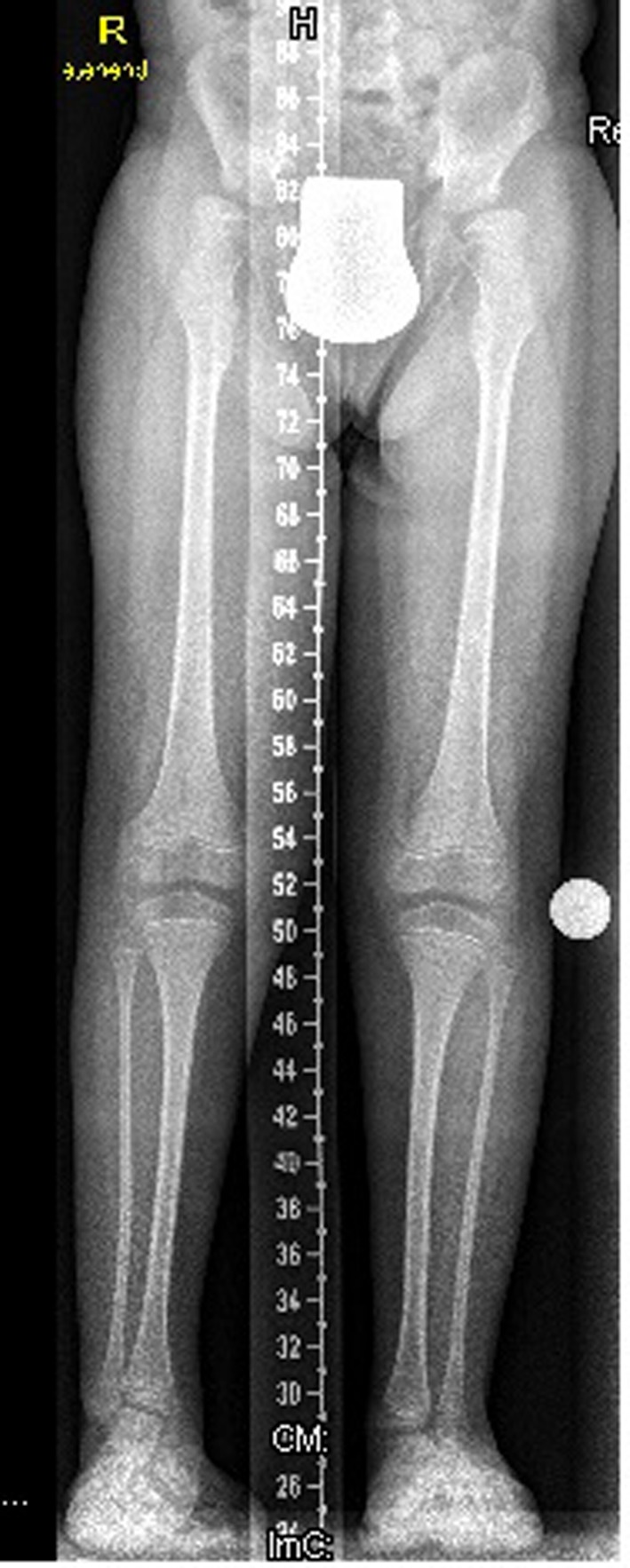
Lower limb standing radiograph showed a hypoplastic iliac bones, severe coxa valga, associated moderate subluxation of the hip because of incomplete development of the acetabulae with over-tubulation of the long bones.

Lower limb standing radiograph showed hypoplastic iliac bones, severe coxa valga, and associated moderate subluxation of the hip because of incomplete development of the acetabulae with over-tubulation of the long bones (Fig. 5).

He was operated to correct his unilateral club foot (right side) through tibialis anterior tendon transfer and the whole tendon was detached after a medial incision and was laterally transferred. A cuboid closing-wedge osteotomy was made and stabilized using a Kirschner wire. The tibialis anterior was transferred thereafter and was pulled through the bone canal to the planta pedis and was sutured to the periosteum. Finally, a percutaneous lengthening of the Achilles tendon associated with mini-invasive plantar fascia release has been applied.

The left foot with vertical talus was treated by performing a percutaneous Achilles tendon lengthening. The latter was followed with an open reduction of the talonavicular joint by application of K wires as an empirical measure to stabilize the reduced situs and finally, capsulorrhaphy of the plantar and medical structures was added.

## Discussion

Orofacial syndrome (OFDS) is characterized by malformations of the face, oral cavity, digits, and central nervous system, and 13 clinical subtypes are delineated. OFD I can be easily distinguished from other subtypes by its X-linked dominant inheritance pattern and is caused by mutations of OFD I encoding a centrosomal protein involved in ciliary function, which is lethal in the male hemizygote. Craniofacially, OFD I is characterized by prominent forehead with flattened basilar kyphosis [3, 6, 12, 13]. The gene is lethal in males, who are thought to die *in utero*, although occasional, severely affected; near term cases are reported [4].

OFD I syndrome is the only form of OFDS for which the molecular is fully elucidated, when the OFD I gene has been identified [14, 15]. Mutations have also been identified in the GLI3 gene in five patients with OFDS and median anomalies including hypothalamic hamartoma/mass, agenesis of corpus callosum, imperforate anus and/or atrial septal defect [15]. Also mutations in the TMEM216 gene, recently reported as responsible of Joubert and Meckel syndromes, have also been identified in two patients with OFD VI syndrome [16, 17].

CNS malformations appear particularly associated with OFD I and VI syndromes. In OFD I syndrome, CNS involvement is reported in about 50% of cases, while corpus callosum agenesis appears relatively common (40%); other malformations, including intracerebral single or multiple epithelial or arachnoid cysts, porencephaly, heterotropias of grey matter, cerebral malformations, abnormal gyrations, and microcephaly, are rarely described in few observations [18].

In OFD VI syndrome, the cerebellar findings of vermis hypoplasia/aplasia, Dandy-Walker anomaly, molar tooth sign together with the recurrence of Y-shaped metacarpals indicating central polydactyly, represent distinctive features thus belonging to the Joubert syndrome related disorders [9, 13].

Agenesis of the pituitary gland, hypothalamic hamartoma with precocious puberty, arhinencephaly, and arachnoid cysts in the posterior fossa are reported in OFD VI syndrome

OFD II (Mohr syndrome) [5] is characterized by a midline cleft of the upper lip, and hypertelorism and micrognathia are also common. The ears may be low-set and/or posteriorly angulated. Bony changes appear limited to hands and feet. Bilateral manual hexadactyly and bilateral polysyndactyly of the halluces are characteristics. The hallucal polysyndactyly in the patients described by Beaudry and Mayer was however unilateral. Christophorou and Nicolaidou [10] reported duplicated thumbs in their patient. Bimanual hexadactyly apparently is not requisite for the diagnosis of the syndrome, since in some cases, there have been five fingers with ulnar deviation of the fifth finger 3-4 syndactyly with extra bones in the web, or hexadactyly of only one hand. Mental retardation has been reported in several cases. Various other neurologic anomalies have been described including microcephaly, porencephaly, internal hydrocephalus, conductive hearing loss, choroid coloboma and muscular hypotonia with poor coordination. Another finding is increased susceptibility to respiratory infections, which in several patients has resulted in death during infancy. Tachypnea has also been reported. Cleft tongue is probably a constant feature of the syndrome and several authors have spoken of general ankyloglossia; in few cases cleft or highly arched palate has been mentioned; however, in most patients, the palate has been intact, a small median cleft of the upper lip is a relatively common feature, but was missing in the case described by Mohr and Claussen [6]. Multiple frenula are occasionally present but far less common than OFD I. Fatty hamartomas on the dorsum have been found in several cases [4, 19]. Christophorou and Nicolaidou [10] also described duplicated thumbs in their patient. Bimanual hexadactyly apparently is not requisite for the diagnosis of the syndrome, and in most cases there have been five fingers with ulnar deviation of the fifth finger, 3-4 syndactyly with extra bones in the web, or hexadactyly of only one hand. Toriello [20] described the heterogeneity and variability in OFDS, sharing oral, facial, and digital anomalies. Based on more or less subtle clinical differences, at least seven causally different entities can be identified in this group. These include: 1) OFDS I; 2) Mohr syndrome (OFDS II); 3) OFDS III; 4) OFDS with tibial anomalies (OFDS IV); 5) Thurston syndrome; 6) Varadi syndrome; and 7) Whelan syndrome.

Hsieh and Hou [21] described the overlapping features of OFD II and OFD VI. Helga [22] described the connection of primary ciliary defects as a causation of ciliary protein mutations.

The combination of post-axial polydactyly with a midline cleft of the upper lip appears to be a condition distinct from the OFDS. Most reports have been in the plastic surgery literature. In some families, there has been autosomal recessive inheritance. Phadke et al [23] reported three sibs from a consanguineous family with overlapping features between OFD type V and Ellis-van Creveld syndrome. Another Indian case was reported by Valiathan et al [24]. Two other sibs were affected. Again, there were multiple frenula as in Ellis-van-Creveld. Also many cases of median cleft of the upper lip are not true median clefts but examples of the median cleft lip form of holoprosencephaly characterized by agenesis of the septum pellucidum and corpus callosum, agensis of the prolabium, premaxilla and nasal bones, microcephaly and hypotelorism true median cleft may be seen in association with bifid nose and hypertelorism in frontonasal malformation.

In summary, distinctive features in our patient include sparse hair, frontal bossing, faint eye brows, hypertelorism, broad nasal root and bifid tip of the nose, midline cleft of the lip/cleft palate and hypertrophied frenula and gingival fibromatosis. The ears are large, low-set and ill-defined modeling. Skeletally he manifested coxa valga, club foot of the right foot and vertical talus of the left foot respectively. Examination of the parents revealed a combination of minor malformations. The overall phenotypic features in our current patient were consistent with the diagnosis of Mohr syndrome but with additional skeletal malformation complex. This is the first report entailing the skeletal and the orthopedic abnormalities in a patient with Mohr syndrome. Further clinical reports are needed to evaluate the degree of the phenotypic variability within the OFD type II spectrum.
